# Efficacy of myofascial trigger point deactivation for tinnitus control

**DOI:** 10.5935/1808-8694.20120028

**Published:** 2015-10-20

**Authors:** Carina Bezerra Rocha, Tanit Ganz Sanchez

**Affiliations:** a PhD. Physical Therapist; b Senior Associate Professor - Otorhinolaryngology Program - Medical School of the University of São Paulo (FM-USP). Department of Otorhinolaryngology - Medical School of the University of São Paulo

**Keywords:** clinical trial, myofascial pain syndromes, tinnitus

## Abstract

Chronic pain in areas surrounding the ear may influence tinnitus.

**Objective:**

To investigate the efficacy of myofascial trigger point deactivation for the relief of tinnitus.

**Method:**

A double-blind randomized clinical trial enrolled 71 patients with tinnitus and myofascial pain syndrome. The experimental group (n = 37) underwent 10 sessions of myofascial trigger point deactivation and the control group (n = 34), 10 sessions with sham deactivation.

**Results:**

Treatment of the experimental group was effective for tinnitus relief (*p* < 0.001). Pain and tinnitus relieves were associated (*p* = 0.013), so were the ear with worst tinnitus and the side of the body with more pain (*p* < 0.001). The presence of temporary tinnitus modulation (increase or decrease) upon initial muscle palpation was frequent in both groups, but its temporary decrease was related to the persistent relief at the end of treatment (*p* = 0.002).

**Conclusion:**

Besides medical and audiological investigation, patients with tinnitus should also be checked for: 1) presence of myofascial pain surrounding the ear; 2) laterality between both symptoms; 3) initial decrease of tinnitus during muscle palpation. Treating this specific subgroup of tinnitus patients with myofascial trigger point release may provide better results than others described so far.

## INTRODUCTION

*Tinnitus* is the perception of sound in the ear or head when no outside sound is present and it occurs in 10 to 15% of the world adult population^1^. The onset of tinnitus might be related to disorders of auditory and/or non auditory systems. For that reason, the association between tinnitus and pain has been the subject of many medical journals recently^2^, ^3^, ^4^. Moreover, some case reports suggested an association between tinnitus and myofascial trigger points (MTP)^5^, ^6^, ^7^, ^8^.

MTPs are small hypersensitive areas located in palpable taut bands of skeletal muscles (muscle, fascia or tendon), which may or not be observed in pain-free subjects, but which are always present in the myofascial pain syndrome^9^. Either spontaneously or under mechanic stimulation, MTP cause local and referred pain with a well-defined pattern for each muscle^10^. Our previous clinical experience showed that patients suffering from myofascial pain syndrome in head and neck regions also complained of tinnitus. After MTP deactivation through digital pressure, patients commonly reported partial or total tinnitus relief in some cases.

Following such clinical evidence, we carried out a previous case control study and found out that tinnitus patients are five times more likely to present MTP, and three times more likely to have a myofascial pain complaint when compared to individuals symptom-free. Furthermore, 55.9% of patients were able to modulate the tinnitus loudness and/or pitch upon MTP palpation^4^. Such patients have been nominated as having somatosensory tinnitus.

Along with our previous experiences, a growing interest in somatosensory tinnitus started and it began to be studied from the standpoint of neural connections between the auditory and somatosensory systems^4^. More than expected, tinnitus was found to be provoked or modulated by stimulation coming from the somatosensory system, similar to those which take place upon MTP palpation^4^, forceful muscle contractions of head, neck and limbs^11^, ^12^ and cutaneous stimulation of the hand/fingertip region^13^.

Some studies have even focused on the evaluation methods for somatosensory tinnitus, but there seems to be a lack of research on treatment yet^14^. Thus, the objective of this paper is to verify the efficacy of MTP deactivation for tinnitus relief in patients with myofascial pain syndrome through (i) investigation of whether tinnitus relief is associated to pain relief; (ii) evaluation of localization and laterality of both symptoms and (iii) inquiry as to whether patients who modulate tinnitus have better prognosis after MTP deactivation.

## METHOD

This study had a double-blind, placebo-controlled, randomized clinical trial design and was previously approved by the Ethics Committee of the institution (CA-PPesq-1383/06) and informed consent was obtained from all subjects.

### Experimental (G1) and control groups (G2)

After carrying out a pilot project, the necessary sample was calculated in 70 individuals, 35 for each group. The inclusion criteria for both groups were: constant or intermittent tinnitus and pain complaint (in head, neck or shoulder girdle) during the previous 3 months; presence of at least one active MTP during physical examination; regular registration in our Tinnitus Research Group between October 2007 and September 2009. Rigid exclusion criteria involved patients with (1) pain complaint involving three or more quadrants of the body, regardless of its cause; (2) injection with local anesthetic and/or specific treatment for MTP deactivation in the past 3 months; (3) use of medication for pain and tinnitus treatment in the past month of evaluation; (4) impossibility of understanding the guidelines set forth and/or providing information on the possible effect of palpation in tinnitus; (5) absence of tinnitus perception at the moment of evaluation (in case of intermittent tinnitus) and (6) pulsatile tinnitus ormyoclonus.

Diagnose criteria for active MTP were: the presence of exquisite tenderness at a nodule in a palpable taut band evoking pain that would correspond to the patient's pre-existing pain complaint^10^. MTP hypersensitiveness was confirmed by the “jump sign” as shown by the patient, which may include withdrawal of the head, wrinkling of the face or forehead or verbal responses^15^. In this research, MTP local twitch response was not a necessary condition for the final diagnose, once this visible muscle contraction is usually observed during sustained palpation or needle introduction^10^.

Palpation was performed with sustained deep single-finger pressure during up to 10 seconds with a spade-like pad at the end of the distal phalanx of the index finger or through pincer palpation (thumb and finger) moving across the muscle band at the hypersensitive area^10^.

All selected subjects underwent:

1. medical screening investigation with the otologist, who would direct patients to the
2. “blind researcher” who investigated the following topics in the before the treatment and after the fifth and tenth treatment sessions:
  a) Tinnitus:
    • General characteristics: 1) duration, 2) location, 3) type: single or multiple, 4) perception: constant or intermittent, 5) subjective loudness (through a numeric scale ranging from 0 to 10, so as to be able to detect immediate temporary changes).
    • Temporary modulation: any immediate increase or decrease in tinnitus loudness (at least one point in the numeric scale) or change on its pitch upon digital pressure of MTP.
    • Validated questionnaire translated to Portuguese dealing with severity (THI - Tinnitus Handicap Inventory)^16^.
  b) Pain:
    • General characteristics and subjective intensity (through a numeric scale ranging from 0 to 10, so as to be able to detect immediate temporary changes) and location (pointed by the subject on a body diagram).
    • Objective evaluation of the pain threshold as well as MTP discomfort through use of pressure algometer, as described by Fischer, calibrated from 0 to 10 kgf/cm^17^.
    • Registration of presence and location of active MTP in body diagrams identifying eight possible muscles: infraspinatus, levator scapulae, superior trapezius, splenius capitis, splenius cervicis, sternal portion of sternocleidomastoid, superficial masseter and anterior temporalis.
  c) Phone call follow-up after two months of the last treatment session: patients from G1 were contacted in order to verify whether their tinnitus remained unchanged and stable ever since (through the numeric scale and THI).
  After the first blind evaluation, patients were eventually directed to the
3. physiotherapist who would randomize all subjects with a coin (“head” would indicate Experimental Group - G1 and “tail”, the Control Group- G2). G1 would be treated for 10 weekly sessions, through MTP pressure followed by myofascial maneuvers in the muscle manipulated, along with guidelines to be followed at home, such as applying heat locally, stretching and postural instructions. It must be pointed out that such guidelines are routine procedures performed in any clinical work during MTP deactivation. Patients of G1 were attended on different days of the G2 in other to avoid contact between the subjects because patients of G2 would undergo a sham deactivation: the physiotherapist placed a finger close to the diagnosed MTP for up to 30 seconds, in a such a way that the pressure is insufficient to deactivate any other MTP around the one which was evaluated.

### Statistical Analysis

Statistical methodology included Fisher's test, *Student's t* test, Mann-Whitney test, Friedman test, *kappa* value and Spearman's rank correlation coefficient, using a significance level of *p <* 0.05.

## RESULTS

In this study 71 subjects were evaluated, 37 of which from G1 and 34 from G2. However, during treatment, 04 patients from G1 and 10 from G2 gave up. Consequently, 33 patients remained in G1 and 24 in G2. There was no significant difference between the groups regarding gender (*p* = 0.499), age (*p* = 0.657), time elapsed since tinnitus (*p* = 0.858) and pain onset (*p* = 0.987), showing that randomization was effective.

In patients from G1: (a) tinnitus annoyance varied from four to 10 in numeric scale; (b) single tinnitus was present in 21 cases and multiple tinnitus in 12 cases (two to seven different sounds in each patient) and (c) tinnitus was constant in 31 patients. In patients from G2 (a) tinnitus annoyance varied from five to eight; (b) single tinnitus was present in 17 cases, and multiple tinnitus in seven cases (two to five different sounds in each patient) and (c) only one patient reported intermittent tinnitus.

Regarding the pain, G1 patients had a discomfort ranging from three to 10 in the numeric scale, 26 had constant pain and seven suffered from intermittent pain. G2 patients reported discomfort ranging from two to eight in the numeric scale, 16 featured constant pain and eight suffered from intermittent pain.

Efficacy of MTP deactivation for tinnitus relief in patients with myofascial pain syndrome and the effect on tinnitus treatment on the medium run (2 months after the end of treatment).

In order to verify efficacy of MTP deactivation in light of tinnitus, we analyzed in both groups: tinnitus intensity, number of sounds (single or multiple sound), total THI and modulation intensity. In all analyzed items, G1 obtained a statistically significant response when compared to G2 (*p* < 0.001).

Tinnitus relief in relation to number of sounds, total THI and modulation intensity showed improvement in the fifth session (*p* < 0.001).

By the end of the treatment, four patients from G1 with constant tinnitus moved on to intermittent tinnitus and two patients with intermittent tinnitus in the first evaluation no longer suffered from the symptom by the tenth session. No change in the frequency of tinnitus was observed in G2.

Regarding the effect of MTP treatment in tinnitus in the medium run, G1 patients showed stable tinnitus scores in the numeric scale and in THI in 75.8% of patients.

### Association between tinnitus relief and pain relief

There was a significant association between pain and tinnitus relieves (*p* = 0.013; Spearman's correlation = 0.426).

While significant improvement in G1 was observed in all tinnitus variables, such improvement was also observed in connection with pain and MTP treatment when G1 was compared to G2 *(p <* 0.001). Responsiveness to MTP treatment with regards to pain intensity and total number of active MTP was better in the tenth session when compared to the fifth *(p <* 0.001).

### Correlation of laterality between tinnitus and pain

In G1, an association of laterality of 54.4% was observed *(kappa =* 0.32; *p <* 0.001) between the ear affected by tinnitus (or the ear with worst tinnitus in bilateral cases) and the side of the body with pain (or the worst pain).

### Tinnitus modulation through MTP palpation

Tinnitus modulation during MTP palpation in the initial evaluation occurred in 25 (75.7%) out of 33 patients from G1 and in 20 (83.3%) out of 24 patients in G2. Both in G1 and in G2, modulation in the intensity of tinnitus was perceived up and down the initial numeric scale, and changes in the type of sound were also observed. The vast majority of patients felt a temporary increase in tinnitus intensity during modulation (72% of G1 and 80% of G2).

A subanalysis in G1 intended to check whether tinnitus relief after MTP deactivation was different between patients who modulated tinnitus and those who did not, but there was no significant difference between them *(p =* 0.081 - Mann-Whitney test). However, analyzing only the patients with modulation d, we noticed that an initial temporary decrease of tinnitus intensity allowed for a significant improvement when compared to those whose tinnitus intensity had initially increased *(p =* 0.002 - Mann-Whitney test).

## DISCUSSION

Previous studies demonstrating a relationship between tinnitus and MTP relied upon local injections of anesthetic in such points^5^, ^6^, ^18^. More patients would benefit from non invasive techniques. In our study, MTP deactivation through digital pressure was deemed effective in each and every tinnitus variable under evaluation and in the medium run responsiveness to treatment remained stable in 75.8% patients. A relevant fact was that tinnitus relief was directly linked to the pain relief. As expected, the MTP deactivation treatment was also effective in G1 when pain intensity, number of MTP and algometer values were analyzed.

Similarities between constant tinnitus and chronic pain are 19, 20, 21:

• both are subjective sensations, present diverse causes, may be influenced by the central nervous system and modulate their intensity throughout the time;
• both have strong psychological component, suggesting that brain areas not directly in charge of sense perception (limbic and autonomic systems) are also involved;
• both auditory and somatosensory systems feature a well-developed network of efferent fibers that seems to have some sort of control over afferent activity.

In favor of such similarities, Rocha^22^ e Rocha & Sanchez^23^ pointed out that patients with tinnitus are three times more likely to present a myofascial pain complaint than individuals without tinnitus. Camparis et al.^2^ maintained that the high prevalence of pain in tinnitus patients is a consequence of sensory-motor interactions observed in individuals with chronic pain and in those complaining of tinnitus.

The correlation of laterality between the ear with tinnitus (or the ear with the worst tinnitus, if bilateral) and the side of the body with pain (or the worst pain) was present in 54.4% *(kappa =* 0,32; *p <* 0.001) of patients from G1. In our previous study, laterality correlation took place in 56.5% *(p <* 0.001) of individuals, mainly in those with pain complaint and tinnitus asymmetry between the ears. Estola-Partanen^18^ also found significant association between the side of the body presenting more muscular tension - related to MTP presence in neck muscles and shoulder girdle - and the ear with worst tinnitus. Bjorne^24^ studied 39 subjects with tinnitus who presented hypersensitive spots in the lateral pterygoid muscle. Among them, 29 had unilateral tinnitus, which coincides with the side of the ear with tinnitus. Travell^5^ and Wyant^6^ have also related that tinnitus-associated MTP would also be located ipsilateral to the symptom. Furthermore, Levine^25^ suggested that somatic stimuli can disinhibit the ipsilateral cochlear nucleus, producing excitatory neuronal activity within the auditory pathway that results in tinnitus.

Anatomical and clinical links between auditory and somatosensory pathways may help understand the influence of myofascial pain on certain kinds of tinnitus, as well as explain how MTP treatment could alleviate the symptom.

The auditory pathway consists of several well-defined centers. The cochlear nucleus is the first central station, receiving information from the cochlear hair cells. While the lemniscal system sends received information to the primary cortical auditory areas, the extralemniscal system transmits them to the associated areas. Neurons of the extralemniscal system also receive information from the somatosensory system, suggesting association between auditory and non-auditory pathways^26^, ^27^.

According to Levine^25^, tinnitus located ipsilateral in relation to the somatic injury rises suspicions over a possible somatosensory component in their origin. In our sample, most patients complained of bilateral tinnitus and reported relief. As a result, one must not forget that tinnitus is a symptom linked to many causes and not infrequently is more than one cause found in the same individual^28^. Consequently, acting upon one of these causes may enhance positive results.

Tinnitus modulation is being researched recently. This clinical phenomenon strongly suggests the existing neural connections between the somatosensory and auditory systems, whose “activation” may play a role in tinni-tus^29^. Tinnitus may be modulated by a variety of muscular stimuli, such as isometric muscular contractions^11^, ^12^, MTP palpation^4^ and tender points^30^.

One of the theories that explain modulation is neuroplasticity. Aberrant crossmodal plasticity seems to play a role in tinnitus induced or modulated by somatosensory stimuli^31^. Abnormal interactions between different sensory networks may contribute to certain aspects of tinnitus. Reorganization or re-mapping of central nervous areas is expected as a normal response to injury^32^. However, it is not possible to predict whether injury-induced plasticity will end up in limited or cross-modal effects, which in turn may result in compensatory or negative effects with pathological changes and unwanted clinical signs^33^. The effects of neural plasticity can generally be divided into early modifications and modifications with a later onset^32^. The remodeling of tonotopic receptive fields within auditory structures (dorsal cochlear nucleus, inferior colliculus, and auditory cortex) seems to be a late manifestation of neural plasticity. Thus, the modulation of tinnitus by stimulating somatosensory might be explained by activating auditory regions through the non-classical pathway^31^.

In our study tinnitus modulation through MTP palpation was very common in both groups, even more than in our last study. This phenomenon was already mentioned by Levine^11^ and Sanchez et al.^12^, who tested tinnitus modulation through isometric contraction maneuvers of head, neck and members, showing tinnitus modulation in 68% and 65.3% respectively, during such contractions. Other studies demonstrated tinnitus modulation by means of stimuli coming from the somatosensory system^13^, ^33^, ^34^, ^35^.

Among our 45 patients from both groups whose tinnitus modulated, 72% from G1 and 80% from G2 reported temporary increase, while others reported decrease in the loudness or change in the type of sound. Such characteristics have also been observed by Levine^11^ and Sanchez et al.^12^ (with muscular contractions) and Bezerra Rocha et al.^4^ (MTP digital pressure). The authors justify this finding through experimental description of a great projection of the cuneate over the cochlear nucleus, with numerous endings rich in glutamate, an excitatory neurotransmitter^29^. Thus, aberrant neuronal activity in auditory pathways of tinnitus patients could be increased through excitatory stimulation of the gracile and cuneate nuclei over dorsal cochlear nucleus, which explains tinnitus increase as the most common effect on patients with some sort of modulation.

Even though many kinds of stimuli may modulate tinnitus, the possible influence of this phenomenon for the prognosis of a therapeutic protocol for tinnitus had never been examined. One of our most remarkable findings was the fact that temporary decrease in tinnitus intensity during MTP palpation, although less common, is more closely related to tinnitus relief by the end of the treatment than the temporary increase in loudness or the change in the type of sound. Such finding warns us of the importance of a diagnosis of tinnitus modulation when it comes to establishing a prognosis for the MTP deactivation treatment.

Moreover, our focus has always been to make an adequate diagnosis and to apply the best customized treatment to each one. Thus, it is of utmost importance to recognize that these findings clearly show that tinnitus onset may be influenced by the existence of pain surrounding the ear, as well as that the tinnitus treatment can be influenced by the pain treatment. We do hope that the tinnitus community can benefit better from including the investigation of pain in the specific anamnesis of tinnitus, as well as from treating it in order to effectively decrease tinnitus as well.

## CONCLUSION

Besides medical and audiological routine investigation, patients with tinnitus should also be evaluated as to: 1) presence of myofascial pain in the vicinity of the ear: head, neck and shoulder girdle; 2) correlation of laterality between both symptoms, and; 3) whether tinnitus intensity diminishes during the modulation test. Consequently, treating these patients' pain through deactivation of MTP along with guidelines to be followed at home may bring consistent tinnitus relief, which in turn, may be stable for at least two months.

## ACKNOWLEDGMENT

Our grateful thanks to the Tinnitus Research Inicia-tive and to CNPq for the financial support, as well as to the examiners Júlia Futaki and Maria Elisabete Pedalini.
